# Ecological prevalence, genetic diversity, and epidemiological aspects of *Salmonella* isolated from tomato agricultural regions of the Virginia Eastern Shore

**DOI:** 10.3389/fmicb.2015.00415

**Published:** 2015-05-07

**Authors:** Rebecca L. Bell, Jie Zheng, Erik Burrows, Sarah Allard, Charles Y. Wang, Christine E. Keys, David C. Melka, Errol Strain, Yan Luo, Marc W. Allard, Steven Rideout, Eric W. Brown

**Affiliations:** ^1^Division of Microbiology, Office of Regulatory Science, Center for Food Safety and Applied Nutrition, U.S. Food and Drug AdministrationCollege Park, MD, USA; ^2^Eastern Shore Agricultural Research and Extension Center, Virginia TechPainter, VA, USA

**Keywords:** *Salmonella* Newport, tomatoes, environmental reservoirs, epidemiological impact, prevalence and diversity

## Abstract

Virginia is the third largest producer of fresh-market tomatoes in the United States. Tomatoes grown along the eastern shore of Virginia are implicated almost yearly in *Salmonella* illnesses. Traceback implicates contamination occurring in the pre-harvest environment. To get a better understanding of the ecological niches of *Salmonella* in the tomato agricultural environment, a 2-year study was undertaken at a regional agricultural research farm in Virginia. Environmental samples, including tomato (fruit, blossoms, and leaves), irrigation water, surface water and sediment, were collected over the growing season. These samples were analyzed for the presence of *Salmonella* using modified FDA-BAM methods. Molecular assays were used to screen the samples. Over 1500 samples were tested. Seventy-five samples tested positive for *Salmonella* yielding over 230 isolates. The most commonly isolated serovars were *S*. Newport and *S*. Javiana with pulsed-field gel electrophoresis yielding 39 different patterns. Genetic diversity was further underscored among many other serotypes, which showed multiple PFGE subtypes. Whole genome sequencing (WGS) of several *S*. Newport isolates collected in 2010 compared to clinical isolates associated with tomato consumption showed very few single nucleotide differences between environmental isolates and clinical isolates suggesting a source link to *Salmonella* contaminated tomatoes. Nearly all isolates collected during two growing seasons of surveillance were obtained from surface water and sediment sources pointing to these sites as long-term reservoirs for persistent and endemic contamination of this environment.

## Introduction

An estimated 48 million illnesses due to the consumption of contaminated food occur annually in the United States. The most common causative bacterial agent of these infections is *Salmonella spp*. (CDC, 2011). Outbreaks of salmonellosis, a gastro-intestinal illness characterized by diarrhea, fever and abdominal cramps, typically manifesting 12–72 h after ingestion, have been associated with melons, sprouts, tomatoes, peppers, mangoes, and leafy greens (Hanning et al., 2009). While food vehicles can be identified it is much harder to pinpoint the source of contamination. Experimental studies have suggested many possible pre-harvest routes of contamination of the produce commodities, such as internalization through the roots, surface contamination of leaves and blossoms, and co-infection with common plant pathogens (Wells and Butterfield, 1997, 1999; Barak and Liang, 2008; Barak et al., 2008; Doyle and Erickson, 2008; Zheng et al., 2013). To compound matters, *Salmonella* is a hardy enteric organism capable of prolonged survival outside of an animal host. Several studies have demonstrated its ability to survive in soil used for planting and fresh water sources used for irrigation and/or pesticide application (Fish and Pettibone, 1995; Winfield and Groisman, 2003; Barak and Liang, 2008; Hanning et al., 2009; Garcia et al., 2010). Post-harvest practices have also been implicated in the spread of contaminants on fresh produce. Dirty equipment, inadequate disinfection of wash water and poor temperature controls are just a few areas identified that can lead to cross-contamination and enrichment of *Salmonella* growth in/on produce commodities (Doyle and Erickson, 2008; Hanning et al., 2009).

Before the 1990's, tomatoes were not considered a typical vehicle for the transmission of *Salmonella* into the human populace. However, a series of outbreaks have propelled tomatoes to one of the most common non-animal food commodities associated with salmonellosis (Hedberg et al., 1999; CDC, 2005, 2007; Bennett et al., 2014). Between 1990 and 2007, at least 12 multistate outbreaks of salmonellosis traced to various types of tomatoes (e.g., red round, Roma, and grape) have been reported, accounting for approximately 1,990 culture-confirmed infections. However, an estimated 97.5% of *Salmonella* infections are not culture-confirmed, signifying that as many as 79,600 illnesses may have occurred during these outbreaks (CDC, 2007). With approximately five billion pounds of fresh market tomatoes eaten in the U.S. annually, the potential for large outbreaks of salmonellosis is cause for concern.

Of the fresh market producers of tomatoes, Virginia ranks third, behind California and Florida (USDA ERS, 2009). Approximately 80% of the vegetables grown in Virginia are grown along the Virginian Eastern Shore (VES), located on the Delmarva Peninsula. Almost yearly, since 2002, an outbreak or incident of *S*. Newport associated with tomatoes grown on the VES has been documented in PulseNet, the Center for Disease Control's molecular subtyping network for bacterial foodborne diseases (Swaminathan et al., 2001). One particular *S*. Newport subtype, PFGE *XbaI* pattern JJPX01.0061, has recurred in 2002, 2005, 2006, and 2010 (CDC, 2007; Bennett et al., 2014). Environmental assessments of the growing fields resulted in the isolation of *S*. Newport, with the same PFGE pattern from irrigation pond water implying that the tomatoes were contaminated pre-harvest (Greene et al., 2008). Unfortunately, the ultimate source of contamination in this environment is not known and only speculative hypotheses are available. Prevalence studies in the local Delmarva growing areas may answer questions as to the distribution and pervasiveness of *Salmonella* in the East Coast U.S. tomato supply and may aid in accounting for infection rates in the mid-Atlantic regions and in other areas of the U.S. that rely on tomatoes cultivated on the VES. To this end, a environmental study aimed at elucidating possible source(s) and reservoir(s) of *Salmonella* contamination of the tomato-growing environment was conducted during the summers of 2010–2011 on Virginia Tech's Agricultural Research Extension Center (AREC) located in the heart of the tomato industry of the VES. This location is a model for the commercial growing fields in the region with access to the same wildlife, insects, native vegetation, and water sources.

## Materials and methods

### Sampling

#### Sampling sites

**Figure 1:**
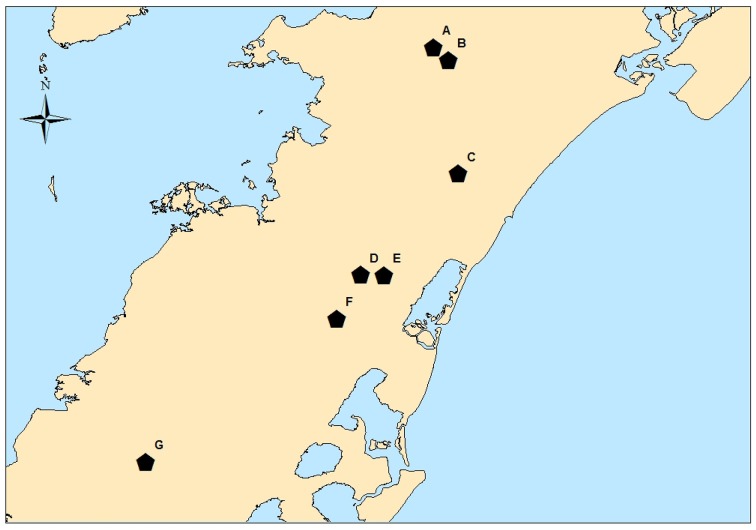
**VES sampling sites**. Map of Virginia Eastern Shore with locations of: various VES waterways (A–F) and AREC (G).

Tomato farm samples were collected from Virginia Tech Eastern Shore AREC in Painter, VA (Figure 1). The farm lies in the heart of VES tomato production and serves as a model for local tomato growing practices. Due to its location, the farm shares similar exposure to wildlife, insects, native weed species, and water sources as the surrounding commercial growing fields. A small plot of tomatoes was planted following recommended good agricultural practices (GAPs) and current industry practices for the local tomato industry with the exception of the application of pesticides since part of the study focused on insects associated with tomato plants. It should be noted, manure is not used by the tomato industry on the VES and it was not used in the model plot of tomatoes. Commercially grown tomatoes were obtained from surrounding fields or purchased at roadside produce stands for analysis.

VES waterways (Figure 1) were selected with input from the Virginia Department of Environmental Quality and were sampled at areas of public access. These samples were added to the study in 2011 after noticing that most isolates in 2010 were recovered from the local creek water and sediment at AREC. To assess if this phenomenon was unique to the AREC or more endemic to the whole of the VES, water and sediments samples were collected in July and September 2011 from six different surface water sites.

#### Sample collection

Farm samples included tomatoes—fruit, flowers, and leaves; fruits, flowers, and leaves of other commodities adjacent to the tomato plants including various cucurbits, peppers, snap beans, and basil; native vegetation surrounding the tomato fields and pond; soil from the field; insects; irrigation water and swabs of irrigation filtration units; pond water and sediment; creek water and sediment; fecal material found within the field and around the pond. All samples were collected aseptically into plastic zip-close bags. Soil and sediment samples were collected using sterile soil core auger bores, where ten 6–12 inch cores were combined per sample. One liter of irrigation water was collected in sterile bottles. Forty liters of pond and creek water were collected in sterile polypropylene carboys. Insects were collected using flytraps, ant traps, yellow and blue sticky cards, yellow pan traps, sweep nets, and malaise traps. Traps placed in the tomato field were left out for approximately 2–4 weeks before collection. Occasionally, net-caught insects were killed in the field using ethyl acetate, however most netted insects were kept alive until right before sample processing. Netted insects were photographed and identified based on morphological characteristics. All samples were transported to FDA facilities at ambient temperatures and stored at 4°C no longer than 72 h until analysis.

### Sample analysis

#### Sample enrichment

All samples were pre-enriched overnight at 35°C in modified buffered peptone water (10 g peptone, 5 g sodium chloride, 7 g disodium phosphate, 3 g monopotassium phosphate per liter) (3M, St. Paul, MN) at a 1:1 (w:v) ratio with a 100 ml minimum volume used for very small samples unless otherwise indicated. Tomato fruits were quartered and then 200 g were placed in sterile Whirlpak filter bags (Nasco, Fort Atkinson, WI) and hand crushed to thoroughly mix. Quartering permitted the analysis of any internal contamination as well as external contamination. Fruits from other commodities were placed in Whirlpak filter bags whole, thoroughly massaged and soaked in pre-enrichment broth. Sample sizes depended on the commodity and maturity of the fruit. All other samples were placed in Whirlpak filter bags and then hand-massaged to thoroughly mix. Sample sizes changed depending on sample type and availability at time of collection, in general sample sizes were 0.5–10 g for blossoms, 10–100 g for leaves, 3–200 g for native vegetation, and 0.3–50 g for feces. Irrigation water was filtered through 0.45 μm mixed cellulose ester (MCE) membrane filters. Filters were then placed in Whirlpak filter bags with 100 ml of pre-enrichment broth. Pond and creek water was concentrated to 250 ml by continuous flow centrifugation (Scientific Methods, Inc., Granger, IN). Twenty-five ml of the concentrated sample was then added to 225 ml pre-enrichment broth in a Whirlpak filter bag. For soil and sediment, a 50 ml conical tube was used to remove 50–130 g from the combined collected sample and pre-enriched in 250 ml broth. The moisture content of the soil influenced the final weight with sediments tending to have higher mass. Individual insects were placed in sterile 50 ml conical tubes, crushed and enriched in 25 ml pre-enrichment broth. Insect cards were placed in Whirlpak filter bags and enriched in enough broth to cover the card. For liquid traps, 25 ml was removed and enriched in 25 ml pre-enrichment broth.

#### Molecular assays

After pre-enrichment all samples were screened using multiplex real-time PCR assays. Three assays were evaluated for specificity since there is no validated method to molecularly screen for salmonellae in foods or environmental samples. Two assays were investigated in 2010 and one assay was assessed in 2011. For the 2010 samples, DNA was isolated by immuno-magnetic separation using the Qiagen BioSpring 96 One-For-All Vet kit (Valencia, CA) following the Animal Tissues and Other Sample Types protocol with the following exceptions: 1 ml of pre-enrichment was centrifuged to collect the cells, which were then resuspended in 100 μl of water and transferred to the lysis plate containing proteinase K (600 mAU/ml, provided with kit). DNA was eluted in 75 μl elution buffer. Two separate triplex assays consisting of two targets specific for *Salmonella* (*invA* and *tsaA*- June and July, or *invA* and *ttrRS* gene region- August and September) and an internal amplification control (IAC) were run on the BioRad CFX96 (Hercules, CA) (Malorny et al., 2004; Gonzalez-Escalona et al., 2009; Hebrard et al., 2009; Deer et al., 2010; González-Escalona et al., 2012). The reactions were run as previously described with 2 μl of sample DNA (Zhang et al., 2011) and the primer/probe pairs listed in Supplemental Table S1. In 2011, a custom lyophilized 4-target multiplex assay designed for use with the 7500 Fast system (Life Technologies, Carlsbad, CA) was used to amplify two *Salmonella* specific targets, *invA* and *apeE*; one *Enterbacteriaceae* target, *gapA*; and an internal positive control (IPC) (Trujilllo et al., 2011; Wong et al., 2011). The assay was run per protocol, starting with DNA isolation from the preenrichments using Applied Biosystems MicroSEQ *Salmonella* spp. Detection Kit without PK (LifeTechnologies). After isolation, 30 μl of DNA was added to the lyophilized assay beads, allowing the beads to dissolve and then centrifuging briefly before thermocycling in fast mode. For all qPCR assays, to ensure that no possible positive cultures were missed, any sample that detected at least one *Salmonella* specific gene was counted as positive and cultured for isolation.

#### Microbial culture

All pre-enrichment cultures, which screened positive by one of the molecular methods, were then cultured for the isolation of *Salmonella* following a modified FDA-BAM method (Andrews et al., 2011). Also a subset of samples from each trip was chosen for *Salmonella* isolation regardless of qPCR screening results. The cultures chosen were samples that have a tendency to cause failures in qPCR and PCR reactions and included but were not limited to- soils, sediments, water, and fecal samples. Modifications made to the FDA-BAM method were as follows: all Rappaport-Vassiliadis (RV) broth tubes were incubated at 42°C. Tetrathionate (TT) broth and RV selective enrichments were plated on Xylose lysine desoxycholate Tergatol-4 (XLT-4) agar, instead of Xylose lysine desoxycholate (XLD) agar, Hektoen enteric (HE) agar with 5 ug/ml novobiocin and Bismuth sulfite (BS) agar. All media were prepared as stated in FDA-BAM (Andrews et al., 2011). TT base, XLT-4 base, HE and BS agars were all purchased from BD Difco (Franklin Lakes, NJ), Tergatol-4 supplement from Oxoid Remel (Lenexa, KS) and novobiocin sodium salt from MP Biomedicals (Solon, OH). Suspect colonies were sub-cultured by streaking for isolation on both XLT-4 and HE with novobiocin to get accurate reactions in isolation on these plates. All suspect isolates that gave appropriate reactions on these media were then verified as *Salmonella* using the Gram-negative bacterial identification card on the Vitek 2 (Biomerieux, Durham, NC). All isolates verified as *Salmonella* were then subjected to further subtype testing including PFGE analysis, molecular serotype determination and traditional serotype determination, if necessary, and whole genome sequencing (WGS) (as indicated). Cultures were preserved at −80°C in BHI broth containing 25% glycerol (final concentration).

### Isolate characterization

#### Serotype determination

Serotype was determined using the CDC standard protocol for the molecular determination of *Salmonella* serotype (CDC, 2009). Briefly, DNA from a pure culture was isolated using Instagene (BioRad). Multiplex PCR was set up using Qiagen HotStar Master Mix (Qiagen), 1 ul of DNA and thermocycled under the following conditions: 95°C, 15 min; 30 cycles of 94°C for 30 s, 48°C for 90 s, 72°C for 90 s; then 72°C for 10 min. DNA from the PCR reactions was then hybridized to the beads with specific O- and H-Ag probes before addition of strepavidin-R-phycoerythrin (Invitrogen div. Life Technologies, Grand Island, NY). After incubation the samples are read using the Bio-Plex instrument (BioRad). Positives are determined based on the ratio of signal to noise using a no template DNA negative control. Serotype is determined based on which antigens are positive for each sample. Traditional serotyping was performed on isolates where neither PFGE analysis nor molecular serotyping could infer a serotype. Traditional serotyping was performed at FDA's Center for Veterinary Medicine, Laurel, MD.

#### Pulse-field gel electrophoresis (PFGE) analysis

PFGE analysis was performed with *XbaI* as outlined in the standard CDC-PulseNet protocol for *Salmonella* (Swaminathan et al., 2001; Ribot et al., 2006). Briefly, after plug preparation and DNA digestion, bands were separated using a CHEF-Mapper (Bio-Rad, Hercules, CA) and the following parameters: separation on 1% agarose gel in 0.5X TBE at 14°C, initial switch time 2.16 s, and final switch time 63.8 s at 6 V/cm gradient for 18–19 h. *Salmonella* Branderup H9812 was used as the size standard reference strain. Upon completion of electrophoresis, gels were stained with ethidium bromide, and images were captured as TIFF files using the Gel Doc XR digital imaging system (Bio-Rad). Band patterns were analyzed using BioNumerics 5.10 (Applied Maths, Sint- Martens-Latem, Belgium), according to the PulseNet protocol (Ribot et al., 2006). One example of each PFGE pattern was submitted to PulseNet to obtain official pattern names and for comparison to relevant clinical cases.

#### Whole genome sequence analysis

A total of 19 strains were included in the whole genome analysis. These strains included four isolated from the summer of 2010 (this paper), nine historical tomato farm isolates from the VES that are part of CFSAN's culture collection, and six clinical isolates from a cluster of illnesses caused by *S*. Newport in the summer of 2010. These clinical isolates were kindly provided by the Departments of Public Heath in Maryland, Virginia and Washington D.C.

WGS was performed as previously described (Allard et al., 2012, 2013; Cao et al., 2013). In brief, genomic DNA was isolated from overnight Trypticase Soy Broth (TSB) cultures of each strain incubated at 37°C using Qiagen DNeasy (Valencia, CA). Shotgun sequencing was performed on the Roche 454 GS Titanium NGS technology (Roche, Branford, CT) (Margulies et al., 2005) to obtain 16-24X coverage high quality draft genomes. The 454 FLX reads for each isolate were mapped to the complete *S*. Newport str. SL254 (CP000013) using the Roche Newbler software package (v 2.5). The draft genomes were annotated using NCBI's Prokaryotic Genomes Automatic Annotation Pipeline (PGAAP) (Klimke et al., 2009). Variable positions were identified as Single Nucleotide Polymorphisms (SNPs) where the site differed from strain SL254 with read depth >=10 and >=95% of the reads contained the SNP. Insertions and deletions were excluded from the SNP analysis given the frequency of homopolymer errors in 454 sequencing (Quince et al., 2009). Once the SNPs of each isolate were identified for the entire set of *S*. Newports, a matrix of mapped base calls for these positions were aggregated into a multiple FASTA format for phylogenetic analysis using GARLI (Zwickl, 2006) with 1000 bootstrap replicates. All GARLI analyses were performed using the GARLI web service (http://www.molecularevolution.org/software/phylogenetics/garli) with the default parameter settings and the GTR+Γ+I nucleotide substitution model. Pairwise distances, as number of differences, were calculated based on the concatenated alignment of 33301 SNPs estimating the diversity among the 19 *S*. Newport strains. The calculation was performed using MEGA5 with 1000 Bootstrap replicates (Tamura et al., 2011). Double substitutions, transitions and transversions, were applied at the same nucleotide site and uniform substitution rates are assumed across sites. Gaps and missing data involved in each pair of sequences in the comparison are ignored. To construct the SNP profile, the total number of SNP positions were plotted as position in the reference chromosome vs. the number of isolates differing at that position from the reference. This plot was done using ggplot2 in R.

#### Accessions

Whole genome shotgun accessions and bioproject accessions are listed in Supplemental Table S2.

### Statistical analysis

For sample categories that were positive for the presence of *Salmonella*, 95% confidence intervals were calculated using a normal approximation to the binomial and the formula 1.96 +/− sqrt(p*5q/n). For sample categories that were negative for the presence of *Salmonella*, a 95% upper tolerance limit was calculated in order to infer the maximum proportion of positive samples in the population that would allow at least a 5% likelihood of seeing zero positive samples given the number of samples taken. Pairwise comparisons between sources positive for *Salmonella* and all other sources were performed using a 2-sided Fisher's Exact Tests in SAS v 9.3 (Cary, NC).

## Results

### Molecular screen for salmonellae

Three multiplex qPCR assays were evaluated as a molecular screen for the detection of *Salmonella*. Two assays were tested in 2010 and one assay was tested in 2011. Table 1 shows the specificity and sensitivity of each assay. For use as a screen, a low false negative (FN) rate is critical to ensure the least likelihood of missing a positive sample because the molecular screen dismissed it as negative. Of the three assays, the *invA*/*ttrRS* assay had the worst FN rate at 100% where it missed all the positive samples. The *invA*/*apeE*/*gapA* assay has the best FN rate, as well as the lowest failure rate amongst the three assays evaluated.

**Table 1:** **Sensitivity and specificity of evaluated qPCR assay's ability to detect salmonellae in environmental sample pre-enrichments**.

**qPCR method**	**Total cultured**	**Sensitivity (%)**	**False negative (%)**	**Specificity (%)**	**False positive (%)**	**Failure rate (%)^d^**
*invA*/*tsaA*^a^	160	50	50	87.8	12.2	3.1
*invA*/*ttrRS*^b^	229	0	100	99.5	0.5	2.6
*invA*/*apeE*/*gapA*^c^	503	66.1	33.9	93.3	6.7	2.2

a *Tested June and July 2010*.

b *Tested August and September 2010*.

c *Tested June through September 2011*.

d *Percent of reactions that failed, i.e., the internal control did not amplify*.

### Prevalence of salmonellae in the VES

**Figure 2:**
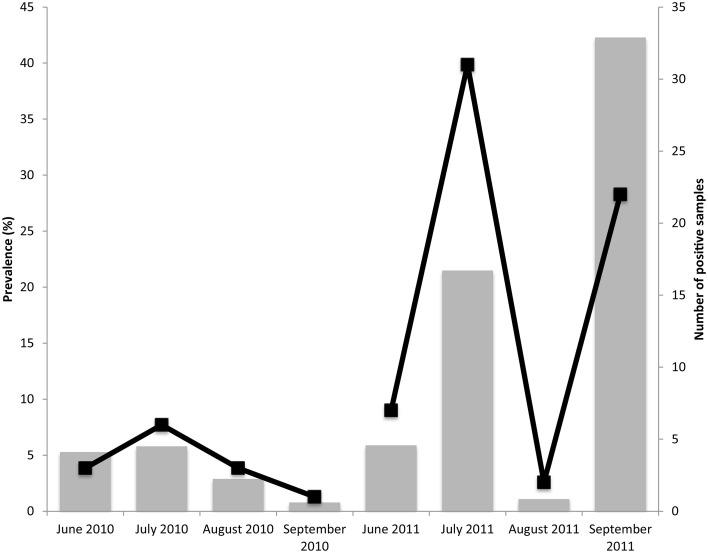
**Prevalence of Salmonella in VES**. Bar and line graph showing prevlance and number of positive samples (respectively) for each month tested. A total of 1570 samples were screened for the presence of *Salmonella*. Overall, 8.4% of the 892 cultured samples were positive for *Salmonella*.

The environmental samples highlighted in Table 2 were collected during the summer months of 2010 and 2011. A total of 1570 samples were analyzed for the presence of *Salmonella*. After the aforementioned molecular screen was applied, 892 of these samples were cultured. Only 75 of the cultured samples were positive for *Salmonella* (8.4% overall prevalence). Figure 2 shows the prevalence for each month sampled. The prevalence was much lower in 2010 (3.3% for the whole summer) than in 2011 (12.3% for the whole summer). It is notable, however, that, for both years, positive sample numbers spiked in July. Among specific reservoirs that were screened regularly during the study, the majority of *Salmonella* positive samples were environmental creek water and creek sediment samples (Table 2), and it was astonishing that no other commodity, insect, native vegetation, farm soil, or well water irrigation samples tested positive throughout the study period.

**Table 2:**
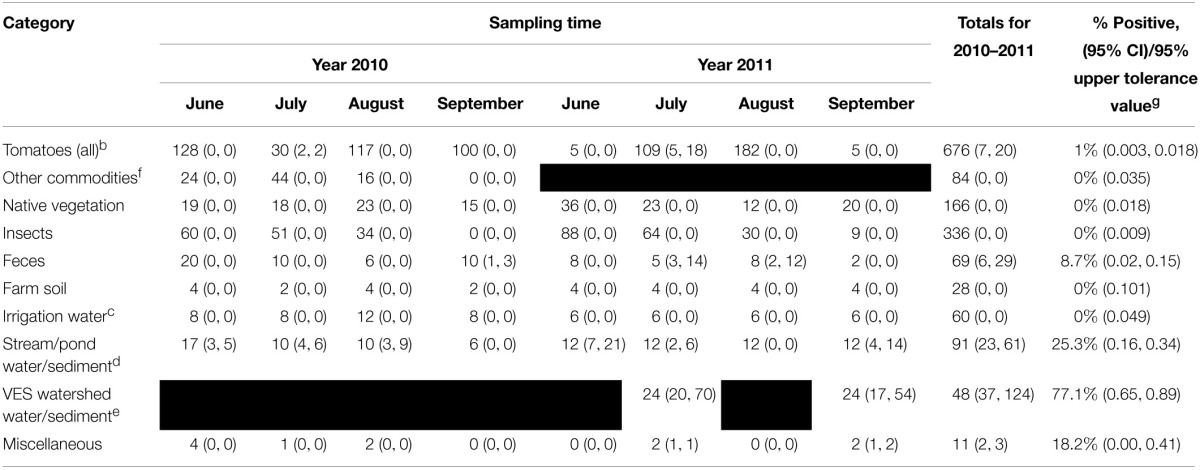
**Total number of samples tested, number positive samples and number of isolates^a^ obtained for 2010–2011**.

*^a^Numbers in parenthesis indicate number of positive samples and number of isolates obtained, respectively*.

*^b^Includes leaves, flowers, and fruits. Samples collected on AREC farm, surrounding commercial fields, and purchased from local farm stands*.

*^c^Includes swabs of irrigation cartridges*.

*^d^Samples from AREC farm only*.

*^e^Collected in July and September 2011 only, from 6 different wadeable streams located within Virginia (see Materials and Methods)*.

*^f^Blackened boxes represent samples not collected during these months*.

*^g^Confidence intervals calculated for categories which tested positive for Salmonella. Tolerance values calculated for categories which tested negative for Salmonella*.

Pairwise comparisons between sources which tested positive for *Salmonella* and all other sources showed VES watershed water/sediment was significantly different from all other sources and the AREC stream/pond water/sediment was significantly different from all other sources with the exception of the miscellaneous sample category. Feces were also significantly different from multiple other sources but not farm soil or miscellaneous (Supplemental Table S3).

### Salmonella isolate identification, diversity, and distribution

**Figure 3:**
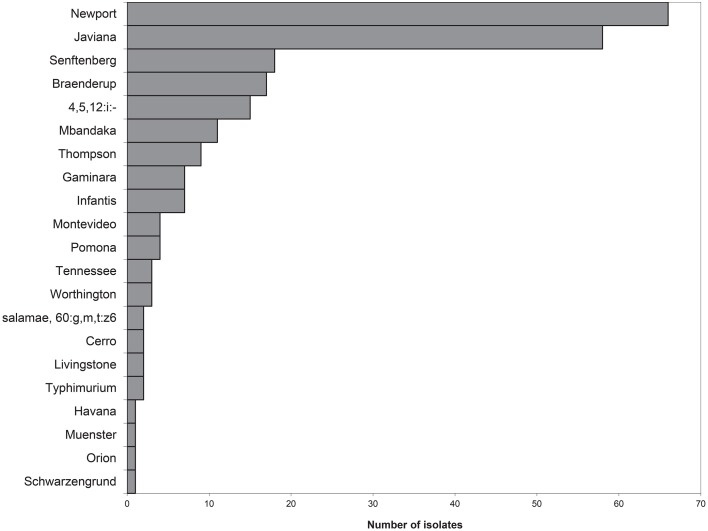
**Serotype distribution of the 234 isolates collected and analyzed, to date, from VES during the summers 2009–2011**. Serotypes were determined by molecular serotyping, inferred from PFGE analysis, or from traditional serotyping. Twenty-one different serotypes were found. *S*. Newport was the most prominent serotype, followed closely by *S*. Javiana.

Over the course of the study, 237 isolates of *Salmonella* were obtained of which 234 were serotyped using the CDC Bioplex method (CDC, 2009) and subsequently subtyped by PFGE. During the characterization of the isolates three were lost. Subspecies and serovar divergence among *Salmonella* isolates was noted with twenty different serotypes of *S*. *enterica* subsp. *enterica* and one *S*. *enterica* subsp. *salamae* found (Figure 3). The most commonly isolated serotype was *S*. Newport closely followed by *S*. Javiana.

**Figure 4:**
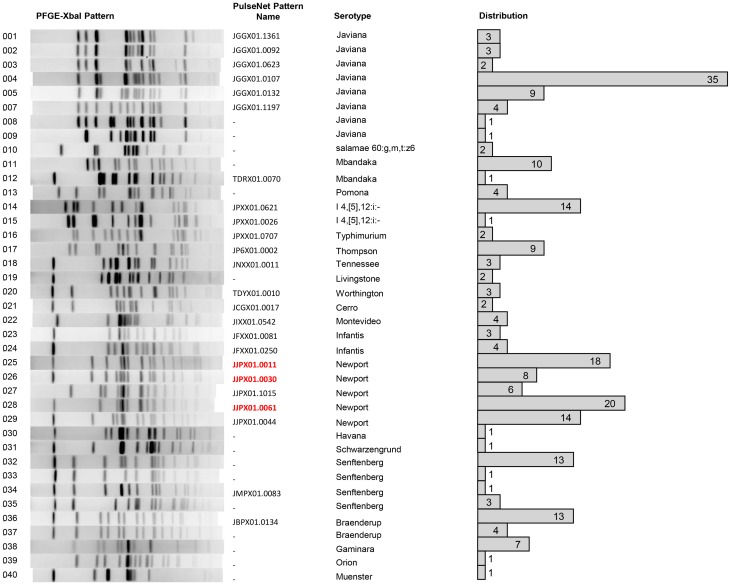
**PFGE analysis**. PFGE was performed on 234 isolates from VES collected during the summers of 2009–2011. PFGE was executed as per standard CDC-PulseNet protocols using *XbaI*. Shown is one representative pattern from isolates with the same pattern along with internal pattern number. Each representative pattern was submitted to PulseNet to obtain an official pattern name, if available. The distribution of each pattern is shown. Many serotypes show more than one PFGE pattern subtype, for example *S*. Javiana has seven different PFGE pattern subtypes.

Each isolate was also analyzed using PFGE with *Xba*I digestion. The PFGE pattern found most commonly was pattern 4, serotype Javiana, with 35 isolates sharing this common fingerprint (Figure 4). *S*. Javiana also had the most diversity among PFGE patterns with a total of eight different patterns seen. This was followed by *S*. Newport with five different patterns and *S*. Senftenberg with four patterns. Other serotypes with multiple patterns included *S*. Mbandaka; *S*. 4,5,12:i:-; *S*. Infantis; and *S*. Braenderup. A representative PFGE pattern from each group was submitted to PulseNet to obtain an official pattern name and to determine whether any of these isolates may have previous clinical relevance. It is noteworthy that several of these patterns had been seen previously in PulseNet. However, 22.6% of study isolates had a unique *Xba*I PFGE pattern, naïve to the national PulseNet database prior to their current submissions. Of the isolates with known patterns, three (pattern 25, 26, and 28) have been linked to tomato-associated foodborne outbreaks. Specifically pattern 28, corresponding to JJPX01.0061, is the recurrent outbreak subtype of *S*. Newport associated with VES tomatoes (Figure 4).

Among all samples collected at AREC, a total of five different serovars with 11 different PFGE subtypes were seen. *S*. Newport was consistently isolated from AREC in 2010 and 2011 (Table 3). In 2010, the same *S*. Newport PFGE subtype, pattern 25, was observed in all *S*. Newport isolated, including those obtained from the creek and from goose feces. However, it is important to note that they were not isolated from both sources within the same month (i.e., from water in June and August; and goose feces in September). In 2011, pattern 25 was only isolated in June at the AREC creek with patterns 26, 27, 28 and 29, observed in later months. Surprisingly, in July of both years, no *S*. Newport was found; however, *S*. Javiana, pattern 5, was seen instead. This was the only time this particular salmonellae was isolated during the study. In July 2011, *S*. Braenderup, with two different PFGE subtypes (patterns 36 and 37), was isolated from the AREC creek and goose feces (Table 3), and this was the only month that this serotype was seen. July 2011 was also the only month in the whole study where *Salmonella* was isolated from tomato fruits. Specifically, *S*. Senftenberg and *S*. Montevideo were isolated from tomatoes collected at two different roadside stands in the region.

**Table 3:** **Distribution of isolates by date, source, serovar and PFGE pattern**.

**Date**	**Location**	**Source**	**Serovar**^a^	**PFGE** **pattern**^b^
June 2010	AREC	Creek water	Newport	25
		Creek sediment	Newport	25
July 2010	AREC	Creek water	Javiana	5
		Creek sediment	Javiana	5
		Tomatoes	60:g,m,t:z6	10
August 2010	AREC	Creek water	Newport	25
		Creek sediment	4,5,12:i:-	15
			Newport	25
September 2010	AREC	Feces	Newport	25
June 2011	AREC	Creek water	4,5,12:i:-	14
			Newport	25, 28
		Creek sediment	4,5,12:i:-	14
July 2011	AREC	Creek water	Braenderup	36, 37
		Creek sediment	Javiana	5
		Feces	Braenderup	36, 37
	Roadside Stand	Tomatoes^c^	Senftenberg	32, 33
		Tomatoes^c^	Montevideo	22
	Site A	Water	Javiana	6
			Mbandaka	11
		Sediment	Infantis	24
			Mbandaka	11
			Thompson	17
	Site B	Water	Infantis	24
			Thompson	17
		Sediment	Mbandaka	11
			Newport	28
	Site C	Water	Javiana	4
		Sediment	Javiana	4
	Site D	Water	Gaminara	38
			Infantis	23
		Sediment	Newport	28
		Algae	Orion	39
	Site E	Water	Gaminara	38
		Sediment	Javiana	4
			Newport	27
August 2011	AREC	Feces	Newport	26, 27, 28
September 2011	AREC	Creek water	Newport	29
		Creek sediment	Newport	29
	Site A	Water	Javiana	9
			Senftenberg	34
			Thompson	17
			Worthington	20
	Site B	Water	Javiana	7, 8
			Mbandaka	11
			Newport	26
			Thompson	17
		Sediment	Cerro	21
			Javiana	7
	Site C	Water	Javiana	1, 4
			Typhimurium	16
		Sediment	Javiana	4
			Typhimurium	16
	Site D	Water	Livingstone	19
			Mbandaka	12
			Schwarzengrund	31
			Senftenberg	35
		Algae	Muenster	40
	Site E	Water	Havana	30
			Javiana	3
			Pomona	13
			Tennessee	18
		Sediment	Pomona	13
	Site F	Water	Javiana	2
		Sediment	Newport	28

a *Most months have multiple isolates of the listed serovar(s)*.

b *PFGE pattern number same as listed in Figure 4*.

c *Tomatoes collected from two different roadside stands, all S. Senftenberg isolates were from one stand, all S. Montevideo isolates were from another stand*.

The greatest diversity in serotype and PFGE subtype was seen among salmonellae isolated collected in the VES waterways. A total of 17 different serovars with 28 different PFGE subtypes were isolated in July and September 2011 across the six different surface water sites (sites A–F, Figure 1). Some sites had very little *Salmonella* diversity, such as site F, which only had two serotypes (Table 3), whereas other sites had six or more serotypes and a variety of PFGE subtypes. Site D, for instance, showed the greatest diversity amongst waterway samples with nine different serovars isolated between July and September. Also, it merits noting that many sites showed greater diversity in September 2011 probably due to sample collection occurring after Hurricane Irene passed directly through the area.

### Whole genomic comparisons of environmental isolates to clinical isolates

**Figure 5:**
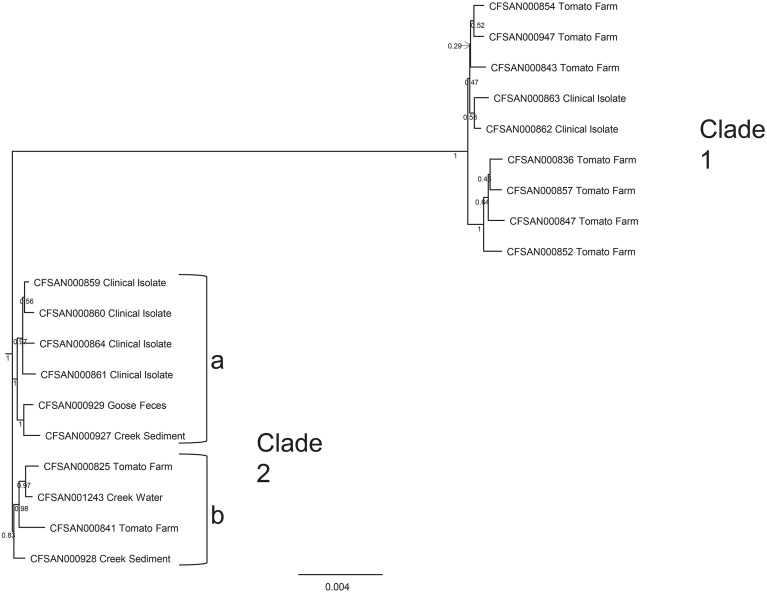
**Whole genome sequence analysis of 19 *S*. Newport strains**. Strains included four isolated from the summer 2010, nine historical tomato farm isolates from the VES that are part of CFSAN's culture collection, and six clinical isolates (kindly provided by the Departments of Public Health in Maryland, Virginia and Washington D.C.) from a cluster of illnesses caused by *S*. Newport in the summer of 2010. Tree was built using GARLI with 1000 bootstrap replicates from a SNP matrix (see Materials and Methods). Two well-supported clades are seen (1 and 2). Clade 1 consists of historical tomato farm isolates and two clinical isolates. Clade 2 consists of historical tomato farm isolates, four VES AREC isolates from 2010 and four clinical isolates. Two AREC isolates, one from goose feces (CFSAN000929) and one from creek sediment (CFSAN000927) (in Clade 2a) have the fewest number of SNP differences from the clinical isolates (CFSAN000859, 000860, 000864, 000861).

**Figure 6:**
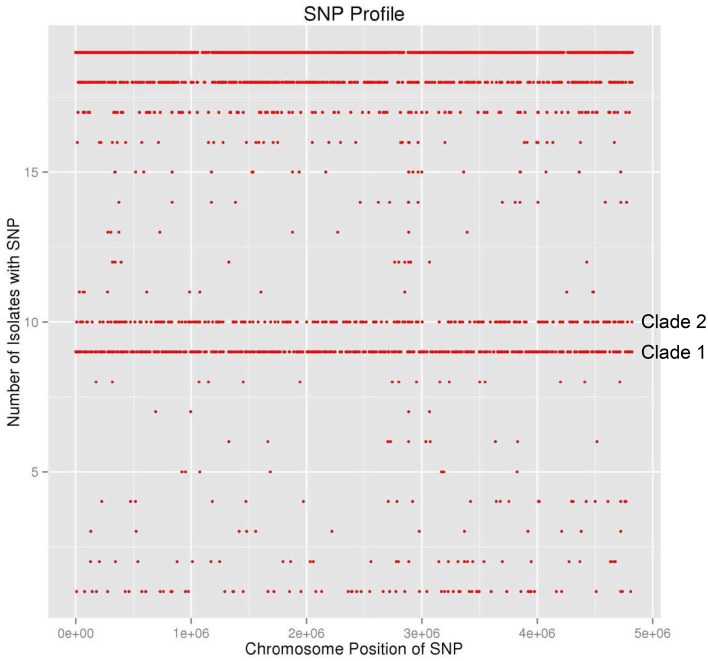
**SNP profile of 19 *S*. Newport strains**. Graphic of SNP position, across the chromosome of *S*. Newport strain SL254, from matrix used to construct the WGS tree (Figure 4). The number of isolates containing the SNP at each position presented on the y-axis. All 19 strains shared 30903 out of 33301 SNPs. All strains in Clade 1 share 671 out of 680 SNPs. All strains in Clade 2 share 288 out of 299 SNPs.

During the summer of 2010, at the same time as the environmental survey reported here was ongoing, a cluster of illnesses, due to *S*. Newport infection, and associated with the consumption of raw tomatoes from one particular establishment, occurred in the Washington D.C. area. This cluster was distinctive in the fact that there were three different *S*. Newport PFGE patterns, namely JJPX01.0061, JJPX01.0011, and JJPX01.0030. Several *S*. Newport isolates collected from AREC had the same PFGE, pattern 25 (JJPX01.0011) (Figure 5), as some of the clinical strains associated with the “D.C. cluster” of illnesses. In order to determine the relatedness of these environmental isolates to the clinical isolates, WGS was applied. In the analysis, along with the clinical isolates from the “D.C. cluster” (CFSAN000859-CFSAN000864) and AREC isolates (CFSAN000927-CFSAN000929, CFSAN001243), several historical *S*. Newport strains isolated from VES tomato farms (CFSAN000825, 000836, 000841, 000843, 000847, 000852, 000854, 000857, 000947) were also included. The whole genome phylogenetic tree revealed several interesting findings (Figure 6). Two distinct well-supported clades were seen. Clade 1 consisted of historical tomato farm isolates and two clinical isolates. All the strains in this clade had a matching PFGE pattern (pattern 28, JJPX01.0061). Clade 2 consisted of historical tomato farm isolates, 2010 AREC isolates, and four clinical isolates. All of the isolates in this clade also had a matching PFGE pattern (pattern 25, JJPX01.0011). The strains with the smallest number of SNP differences to the clinical isolates in Clade 2a are two isolates from AREC, one from goose feces isolated in September 2010 (CFSAN000929) and one from creek sediment (CFSAN000927) isolated in August of the same year. These two AREC isolates appear to be sisters with very high bootstrap support. The other AREC isolates, one from creek water isolated in June (CFSAN001243) and one from creek sediment (CFSAN000928) also isolated in June are in a separate subclade (Clade 2b). Interestingly, even though creek water and sediment samples were collected from the same site, they are genomically quite different from June to August. Intraclade distances are quite small (81 for Clade 1 and 75 for Clade 2) and are much smaller than the interclade distance of 1026 (Supplemental Table S4). An examination of the SNP profile showing the number of isolates containing a SNP (defined as a change from the reference sequence at a particular position) show that the SNPs are located evenly across the whole chromosome (Figure 6). All isolates share 30903 out of 33301 SNPs. Clade 1 isolates share 671 out of 680 SNP positions and Clade 2 isolates share 288 out of 299 SNP positions. Taken together, the whole genome sequence data reported here underscore the importance of a complementary and temporal environmental microbiological sampling to enhance the traceability of produce-borne contamination events back to source.

## Discussion

For surveillance studies, where large numbers of samples need to be tested for the presence/absence of a pathogen, molecular screening assays are desirable. Unfortunately, no validated molecular methods for the detection of *Salmonella* in environmental samples exist so methods that were currently under development were evaluated. Assays used in 2010 were found to be unreliable with very high FN rates. However, the screen used in 2011 proved to be more reliable with an improved FN rate and fewer failures when tested on the environmental samples reported in this study. None of the assays evaluated gave sensitivity or specificity values in the desired range (i.e., close to 100% for both). Problems with these methods highlight the difficulties associated with the development of molecular methods to screen for *Salmonella*, especially in food and environmental samples.

The overall prevalence of *Salmonella* positive cultures from our environmental survey was 8.4%. This was higher than the prevalence reported previously by Micallef et al. (2012) of 1.1% for samples taken from tomato farms on the VES during 2009–2010. This was also higher than the prevalence found in California during a survey in 2008–2009 and in New York from 2009 to 2011 (2.3% and 4.6%, respectively) (Gorski et al., 2011; Strawn et al., 2013). Similar to other studies, however, surface water and sediment were the most common sources of *Salmonella* positives, albeit the prevalence reported here was much greater than other surveys [43.2% of the surface water/sediment samples here, 3.3% Micallef et al. (2012), 7.1% Gorski et al. (2011), and 9.0% Strawn et al. (2013)]. Rather, prevalence of *Salmonella* positive water and sediment samples was similar or lower than other surveys focused specifically on environmental surface waters in California, Florida, Georgia and North Carolina (30.7%, 100%, 79.2% and 54.7%, respectively) (Haley et al., 2009; Patchanee et al., 2010; Rajabi et al., 2011; Walters et al., 2011). Differences observed between this study and the one conducted by Micallef et al. (2012) may reflect differences in isolation methods as well as specific sampling areas especially with respect to water samples. Additionally, the farm ponds sampled by Micallef et al. (2012) may have been isolated and recharged from well water or from field run-off only, whereas water samples collected here were from small streams that would receive run-off from a mixture of sources including farm fields, rural/human areas, and animal industry and wildlife. These sources could contribute to a higher level of *Salmonella* contamination.

Thirty-nine different PFGE patterns were seen within the 21 *Salmonella* serovars. Twenty-four of these patterns had previously been uploaded to PulseNet and have been named. The remaining 15 were novel and have not been named by PulseNet. Thus, the majority of the isolates are clinically relevant, some with the same PFGE patterns as recent outbreak clusters (CDC, 2007; Greene et al., 2008; Bennett et al., 2014; Angelo et al., 2015). Isolates with unique PFGE patterns (22% of the isolates) are intriguing and can be accounted for in two ways. First, these isolates could represent salmonellae that are more highly adapted to persist in non-human host/environments. Alternatively, these variants could represent emerging pathogens that have yet to cause a significant number of illnesses for detection by PulseNet. WGS should be able to elucidate genomic and phylogenetic relationships of these isolates, which are not yet clinically significant, to other strains that are more clinically relevant. Despite these differences, it is important to note that all of the isolates collected are capable of infecting humans and their prevalence should continue to be monitored, particularly given their proximity to commercial sources of tomatoes and other crop commodities.

The two most prevalent serovars with the most PFGE pattern diversity were *S*. Newport and *S*. Javiana. Both of these serovars have been associated with tomato related outbreaks (CDC, 2005, 2007; Greene et al., 2008; Bennett et al., 2014). The majority of *S*. Newports isolated in this study were PFGE pattern 28 (JJPX01.0061) which has been associated with recurrent outbreaks linked to tomatoes grown on the eastern shore since 2002 and up to as recently as 2014 (Greene et al., 2008; Bennett et al., 2014; Angelo et al., 2015). Patterns 25 (JJPX01.0011) and 26 (JJPX01.0030) have also been connected to tomato-related illnesses and were isolated in high frequency. Interestingly, the remaining *S*. Newport subtypes, patterns 29 (JJPX01.0044) and 27 (JJPX01.1015), are relatively rare patterns in PulseNet with only five or three entries in the database (respectively) over the past 2 years. *S*. Javiana showed the most PFGE subtype diversity, with seven different patterns. Also of note, one of the seven *S*. Javiana subtypes collected from VES had not been seen previously in PulseNet, indicating a potentially newly evolved strain, or at the very least, a very recent introduction into an agriculturally active region.

The only *Salmonella* isolates obtained from tomato fruit were collected in July 2011 from tomatoes purchased at two different roadside stands. From one roadside stand three samples resulted in the detection of 14 isolates of *S*. Senftenberg. Of these isolates, two different PFGE patterns were observed. It was unexpected that neither of these two tomato-related PFGE subtypes was seen in *S*. Senftenberg detected in water from two different creeks in September 2011. At the second roadside stand, *S*. Montevideo was isolated from two samples yielding four different isolates. All of these isolates had the same PFGE pattern but *S*. Montevideo was not found in any other sample collected along the VES.

Geese are known to be carriers of *Salmonella* without showing signs of infection (Feare et al., 1999; Fallacara et al., 2004; Christensen et al., 2011). Because of this, many hypothesize that the source of the *Salmonella* contamination in the environment is due to the large population of resident and migratory geese on the VES. During this survey, *Salmonella* was isolated from five samples of goose feces (1 in Sept 2010, 3 in July 2011 and 1 in Aug 2011). This is in contrast to the study done by Gruszynski et al. (2013) where none of the geese samples tested positive for *Salmonella*. This may be due to the fact that the group collected only seven goose samples during their 9 month study period. If the goose populations were indeed responsible for the endemic environmental contamination levels seen in the VES, fecal samples should have yielded *Salmonella* positives each month and in far more of the collected samples. While it is possible that *Salmonella* died off in the feces before collection, this seems unlikely. *Salmonella* has been shown to survive for at least 4 weeks in chicken litter amended soils (Islam et al., 2004; Nyberg et al., 2010) and up to 28 days in geese feces (Feare et al., 1999) and, in this study, samples were collected as fresh as possible and nearly always prior to any significant dehydration. These data do suggest, however, that geese may acquire *Salmonella* from one water source and deposit it at a different water source. For instance, during the summer 2010, *S*. Newport pattern 25 was seen in the water and sediment at AREC in June and August. This same subtype was also seen in geese feces in September 2010 suggesting that the goose had acquired the *Salmonella* from the water and perhaps served as a reservoir for this subtype to overwinter to June 2011 when it was seen in the creek water at AREC. This relationship between the geese and water is also seen in July 2011 when *S*. Braenderup was isolated from the creek water at AREC and three samples of goose feces collected at AREC. Gulls have also been proposed as possible carriers of *Salmonella* to tomatoes while in the field (Gruszynski et al., 2014). However, no sample from seagull dropping tested positive for the presence of *Salmonella* and rarely were seagulls observed around the tomato plants while in the field. Similar to geese, gulls may act as carriers of *Salmonella* from the sources of contamination, such as poultry processing plants or landfills, to surface waters that could be used for irrigation of or pesticide application to tomato plants. Thus, adding to the endemic contamination of this isolated environment. Additional studies focused on potential wildlife and other animal reservoirs of *Salmonella* in this environment should further define the role, if any, of geese and gulls that amass in this part of Virginia during the tomato-growing season.

The reporting here of a high-resolution genomic linkage of strains isolated from VES to clinical isolates associated with a 2010 Mid-Atlantic tomato-associated foodborne illness event highlights the power of combining real-time monitoring for *Salmonella* on and around farm environments with whole-genome sequencing strain typing technology. As noted in previous reports (Allard et al., 2012, 2013), this novel approach can provide invaluable insight when supporting epidemiological traceback of foodborne outbreak events. In the example reported here, significance lies in the fact that isolates responsible for human illness were isolated at the same time that VES tomatoes were in the field; strongly indicting surface water as the source of contamination. It is also noteworthy that this analysis, although retrospective in nature, provided FDA investigators with a key environment and geographical link at a time when previous investigatory efforts had fallen inconclusive for this event.

Taken together, these results reveal environmental surface water sources as a reservoir for *S*. Newport in the VES ecosystem. *S*. Newport was isolated from creek water and creek sediment from multiple locations across the VES as well as waterfowl feces adjacent to surface waters. While water appears to be a major reservoir for *Salmonella* and may be a contributing factor to tomato contamination, the vector of transmission or means by which contaminated surface waters are transferred to tomato plants can still only be hypothesized. The isolation, however, of an *S*. Newport strain from an irrigation pond run-off stream that nearly matched an outbreak strain by whole-genome sequencing suggested that it is possible for pond water to contribute to *Salmonella* contamination of tomatoes even when drip irrigation and plasticulture are used for tomato production. Indeed, it is reasonable to envision that use of contaminated water to irrigate tomato fields or to mix pesticides for direct application to tomato plants could lead to contaminated fruit; especially in light of previous work showing *S*. Newport contaminated irrigation water was able to move directly into tomato roots, stems, and fruits (Hintz et al., 2010). It is important to note, that spot inoculation of tomato flowers leads to an astonishing rate of internalized *Salmonella* contaminated tomato fruits (Guo et al., 2001; Zheng et al., 2013) with *S*. Newport retaining the greatest levels of relative fitness when compared to four other *Salmonella* serovars in tomato rhizosphere and phyllosphere (Zheng et al., 2013). Furthermore, quite remarkable was the demonstration that *S*. Newport was the only serovar found to internally contaminate a tomato fruit following transplant and after inoculation of the surrounding soil (Zheng et al., 2013).

Identifying the original source(s) of environmental contamination of the VES with *Salmonella* remains key to mitigating produce safety in the region. Clearly, water and sediments within the VES watershed serve as reservoirs for important, clinically-relevant isolates of *Salmonella* including *S*. Newport and these waters may serves as a potential source for the direct contamination of tomato plants. It is notable that *Salmonella* serovar diversity was greatest among isolates collected from creeks in the Northern part of the VES peninsula, near the top of the watershed. This upper region of the VES retains concentrated animal industry (i.e., poultry processing) and stands in geographic contrast to much of the concentrated tomato production environs largely centered south of this point and located downstream. Future studies that include larger sampling areas (e.g., Northern VES and other sites along the Delmarva Peninsula) and incorporate genomic relatedness studies on the salmonellae described here should clarify the geo-spatial relationships among many of these isolates and surrounding sources of potential environmental contamination as well as provide clues for the future traceback of tomato-associated illnesses attributed to this critically important agricultural production region of the United States.

### Conflict of interest statement

The authors declare that the research was conducted in the absence of any commercial or financial relationships that could be construed as a potential conflict of interest.
