# Supplementary material for: Ecological prevalence, genetic diversity, and epidemiological aspects of Salmonella isolated from tomato agricultural regions of the Virginia Eastern Shore
Source: Front Microbiol. 2015 May 7;6:415. doi: 10.3389/fmicb.2015.00415 (PMC4423467; doi:10.3389/fmicb.2015.00415)
Supplement: Supplementary file 1 [file Table1.DOC]

Table S1. Primers used in PCR analysis

| **PCR Method** | **Primers** | **Probes** | **Final Concentration** | **Sequence (5’→ 3’)** |
| --- | --- | --- | --- | --- |
| Conventional multiplex PCR | invA F1 |  | 0.4 uM | CTGCTTTCTCTACTTAACAGTGCTCG |
| Conventional multiplex PCR | invA R1 |  | 0.4 uM | CGCATCAATAATACCGGCCTTC |
| Conventional multiplex PCR | apeE F2 |  | 1.6 uM | TTTGCCGATCATTTACATCCC |
| Conventional multiplex PCR | apeE R3 |  | 1.6 uM | CCAGAGAACCGGCTATCAATCC |
| Conventional multiplex PCR | gapA F1 |  | 1.6 uM | CATTGTTTTCCGTGCTGCTCAG |
| Conventional multiplex PCR | gapA R3 |  | 1.6 uM | AGCATCGAACACGGAAGTGC |
| 2010 qPCR^1^ | invA_176F |  | 0.2 uM | CAACGTTTCCTGCGGTACTGT |
| 2010 qPCR^1^ | invA_291R |  | 0.2 uM | CCCGAACGTGGCGATAATT |
| 2010 qPCR^1^ |  | invA_tx_208P | 0.15 uM | TX-CTCTTTCGTCTGGCATTATCGATCAGTACCA-BHQ2 |
| 2010 qPCR^2^ | tsaA_F |  | 0.2 uM | GAACTCAGAGTCGAAAGA |
| 2010 qPCR^2^ | tsaA_R |  | 0.2 uM | CTGATCGCCTTTGATAAAC |
| 2010 qPCR^2^ |  | tsaA_FAM | 0.15 uM | FAM-ACGCCGACCACTTCAACG- Iowa Black FQ |
| 2010 qPCR^3^ | ttr-6F |  | 0.2 uM | CTCACCAGGAGATTACAACATGG |
| 2010 qPCR^3^ | ttr-4R |  | 0.2 uM | AGCTCAGACCAAAAGTGACCATC |
| 2010 qPCR^3^ |  | ttr-5P | 0.15 uM | FAM- CACCGACGGCGAGACCGACTTT-BHQ3 |
| 2010 qPCR^1^ | IAC-F |  | 0.1 uM | CTAACCTTCGTGATGAGCAATCG |
| 2010 qPCR^1^ | IAC-R |  | 0.1 uM | GATCAGCTACGTGAGGTCCTAC |
| 2010 qPCR^1^ |  | IAC-Cy5P | 0.15 uM | Cy5-AGCTAGTCGATGCACTCCAGTCCTCCT-Iowa BlackRQ-Sp |

^1^Primer and probe set used in all 2010 qPCR reactions

^2^Primer and probe set used in June and July 2010 qPCR reactions only

^3^Primer and probe set used in August and September 2010 qPCR reactions only
