# Supplementary material for: Ecological prevalence, genetic diversity, and epidemiological aspects of Salmonella isolated from tomato agricultural regions of the Virginia Eastern Shore
Source: Front Microbiol. 2015 May 7;6:415. doi: 10.3389/fmicb.2015.00415 (PMC4423467; doi:10.3389/fmicb.2015.00415)
Supplement: Supplementary file 2 [file Table2.DOC]

Table S2. Accession numbers (pending)

| **Strain ID** | **NCBI WGS Accession #** |
| --- | --- |
| CFSAN000825 | AJMN00000000 |
| CFSAN000836 | AJMO00000000 |
| CFSAN000841 | APGQ00000000 |
| CFSAN000843 | APGR00000000 |
| CFSAN000847 | APGS00000000 |
| CFSAN000852 | APGT00000000 |
| CFSAN000854 | APGU00000000 |
| CFSAN000857 | APGV00000000 |
| CFSAN000859 | APGW00000000 |
| CFSAN000860 | APGX00000000 |
| CFSAN000861 | APGY00000000 |
| CFSAN000862 | APGZ00000000 |
| CFSAN000863 | APHA00000000 |
| CFSAN000864 | APHB00000000 |
| CFSAN000927 | APHC00000000 |
| CFSAN000928 | APHD00000000 |
| CFSAN000929 | APHE00000000 |
| CFSAN000947 | APHF00000000 |
| CFSAN001243 | APHG00000000 |
