# Supplementary material for: Ecological prevalence, genetic diversity, and epidemiological aspects of Salmonella isolated from tomato agricultural regions of the Virginia Eastern Shore
Source: Front Microbiol. 2015 May 7;6:415. doi: 10.3389/fmicb.2015.00415 (PMC4423467; doi:10.3389/fmicb.2015.00415)
Supplement: Supplementary file 3 [file Table3.DOCX]

Table S3. Pairwise^a^ comparison between sources that tested positive for the presence of *Salmonella* vs. all other sources

|  | Sources Tested Positive for *Salmonella* | | | | |
| --- | --- | --- | --- | --- | --- |
|  | Tomatoes | Feces | Stream/Pond Water Sediment | VES Watershed Water/Sediment | Miscellaneous |
| Tomatoes |  | 0.0072 | 0.0001 | 0.0001 | 0.0079 |
| Other Commodities | NS 1.0 | 0.0074 | 0.0001 | 0.0001 | 0.0123 |
| Native Vegetation | NS 0.356 | 0.0005 | 0.0001 | 0.0001 | 0.0035 |
| Insects | NS 0.1027 | 0.0001 | 0.0001 | 0.0001 | 0.0009 |
| Feces | 0.0072 |  | 0.0072 | 0.0001 | NS 0.3021 |
| Farm Soil | NS 1.0 | NS 0.1772 | 0.0018 | 0.0001 | NS 0.0742 |
| Irrigation Water | NS 1.0 | 0.0299 | 0.0001 | 0.0001 | 0.0221 |
| Stream/Pond Water Sediment | 0.0001 | 0.0072 |  | 0.0001 | NS 0.2717 |
| VES Watershed Water/Sediment | 0.0001 | 0.0001 | 0.0001 |  | 0.0005 |
| Miscellaneous | 0.0079 | NS 0.3021 | NS 0.2717 | 0.0005 |  |

^a^Pairwise comparisons calculated using a 2-sided Fisher’s Exact Tests in SAS v 9.3 (Cary, NC).
