# Supplementary material for: Ecological prevalence, genetic diversity, and epidemiological aspects of Salmonella isolated from tomato agricultural regions of the Virginia Eastern Shore
Source: Front Microbiol. 2015 May 7;6:415. doi: 10.3389/fmicb.2015.00415 (PMC4423467; doi:10.3389/fmicb.2015.00415)
Supplement: Supplementary file 4 [file Table4.DOCX]

|  | CFSAN000854 | CFSAN000947 | CFSAN000843 | CFSAN000863 | CFSAN000862 | CFSAN000836 | CFSAN000857 | CFSAN000847 | CFSAN000852 | CFSAN000859 | CFSAN000860 | CFSAN000864 | CFSAN000861 | CFSAN000929 | CFSAN000927 | CFSAN000825 | CFSAN001243 | CFSAN000841 | CFSAN000928 |
| --- | --- | --- | --- | --- | --- | --- | --- | --- | --- | --- | --- | --- | --- | --- | --- | --- | --- | --- | --- |
| CFSAN000854 |  | 6 | 7 | 6 | 5 | 7 | 9 | 6 | 10 | 33 | 31 | 30 | 31 | 32 | 34 | 30 | 30 | 33 | 27 |
| CFSAN000947 | 41 |  | 6 | 8 | 5 | 7 | 9 | 7 | 7 | 35 | 31 | 33 | 33 | 32 | 35 | 32 | 31 | 34 | 28 |
| CFSAN000843 | 66 | 64 |  | 7 | 8 | 9 | 11 | 7 | 10 | 34 | 31 | 32 | 33 | 30 | 32 | 32 | 31 | 33 | 28 |
| CFSAN000863 | 65 | 55 | 77 |  | 7 | 5 | 6 | 7 | 8 | 33 | 30 | 29 | 31 | 32 | 34 | 30 | 30 | 32 | 28 |
| CFSAN000862 | 59 | 56 | 74 | 54 |  | 5 | 8 | 8 | 12 | 32 | 31 | 30 | 33 | 33 | 37 | 30 | 30 | 33 | 28 |
| CFSAN000836 | 84 | 79 | 104 | 89 | 91 |  | 5 | 8 | 7 | 32 | 31 | 30 | 30 | 30 | 32 | 29 | 29 | 32 | 26 |
| CFSAN000857 | 88 | 86 | 108 | 104 | 99 | 55 |  | 5 | 6 | 35 | 32 | 31 | 34 | 34 | 35 | 32 | 31 | 33 | 30 |
| CFSAN000847 | 98 | 96 | 112 | 108 | 102 | 69 | 76 |  | 6 | 33 | 30 | 31 | 31 | 31 | 33 | 29 | 30 | 31 | 27 |
| CFSAN000852 | 85 | 86 | 98 | 88 | 95 | 64 | 62 | 72 |  | 36 | 33 | 32 | 34 | 35 | 36 | 32 | 32 | 34 | 32 |
| CFSAN000859 | 1010 | 1002 | 1022 | 1006 | 1001 | 1028 | 1031 | 1032 | 1040 |  | 6 | 6 | 7 | 6 | 7 | 8 | 6 | 9 | 6 |
| CFSAN000860 | 1012 | 997 | 1026 | 1005 | 1010 | 1021 | 1032 | 1037 | 1034 | 41 |  | 7 | 6 | 8 | 5 | 7 | 7 | 7 | 8 |
| CFSAN000864 | 1018 | 1012 | 1029 | 1014 | 1008 | 1037 | 1039 | 1037 | 1039 | 43 | 50 |  | 6 | 6 | 5 | 8 | 8 | 6 | 7 |
| CFSAN000861 | 1019 | 1007 | 1029 | 1011 | 1013 | 1033 | 1040 | 1040 | 1043 | 50 | 44 | 54 |  | 7 | 6 | 10 | 9 | 10 | 10 |
| CFSAN000929 | 1010 | 1004 | 1022 | 1011 | 1010 | 1033 | 1033 | 1034 | 1036 | 69 | 67 | 78 | 74 |  | 6 | 8 | 6 | 9 | 6 |
| CFSAN000927 | 1020 | 1013 | 1027 | 1023 | 1025 | 1040 | 1044 | 1046 | 1041 | 72 | 66 | 88 | 75 | 64 |  | 8 | 7 | 10 | 5 |
| CFSAN000825 | 1013 | 1005 | 1029 | 1017 | 1016 | 1034 | 1036 | 1036 | 1046 | 79 | 73 | 90 | 88 | 94 | 94 |  | 4 | 10 | 10 |
| CFSAN001243 | 1009 | 1002 | 1024 | 1010 | 1002 | 1030 | 1028 | 1021 | 1030 | 77 | 75 | 83 | 83 | 82 | 98 | 51 |  | 7 | 10 |
| CFSAN000841 | 1049 | 1041 | 1062 | 1048 | 1047 | 1070 | 1074 | 1072 | 1077 | 114 | 81 | 85 | 86 | 88 | 96 | 104 | 62 |  | 8 |
| CFSAN000928 | 1002 | 991 | 1013 | 1005 | 1002 | 1018 | 1024 | 1028 | 1022 | 69 | 70 | 73 | 76 | 78 | 88 | 75 | 65 | 74 |  |

Table S4. Pairwise distances^a,b^ of the 19 strains used for WGS analysis.

^a^The number of base differences per sequence from between sequences are shown. Standard error estimate(s) are shown above the diagonal. The analysis involved 19 nucleotide sequences. All ambiguous positions were removed for each sequence pair. There were a total of 36920 positions in the final dataset. Evolutionary analyses were conducted in MEGA5*.

^b^Intra- and Interclade distance were calculated based on mean distance for all strains within and between the clades. Clade 1 intraclade distance is 81, Clade 2 intraclade distance is 75. Interclade distance between Clade I and Clade II is 1026.

*Tamura K., Peterson D., Peterson N., Stecher G., Nei M., and Kumar S. (2011). MEGA5: Molecular Evolutionary Genetics Analysis using Maximum Likelihood, Evolutionary Distance, and Maximum Parsimony Methods. Molecular Biology and Evolution 28: 2731-2739.
